# A qualitative study of perceived barriers to hepatitis C care among people who did not attend appointments in the non-urban US South

**DOI:** 10.1186/s12954-020-00409-9

**Published:** 2020-09-18

**Authors:** Jacqueline E. Sherbuk, Alexa Tabackman, Kathleen A. McManus, Terry Kemp Knick, Julie Schexnayder, Tabor E. Flickinger, Rebecca Dillingham

**Affiliations:** 1 Division of Infectious Diseases and International Health, Department of Medicine, University of Virginia, Charlottesville, VA USA; 2 University of Virginia School of Medicine, Charlottesville, VA USA; 3 Division of General, Geriatric, Palliative, and Hospital Medicine, Department of Medicine, University of Virginia, Charlottesville, VA USA

**Keywords:** Hepatitis C, Linkage to care, Content analysis, Substance use, Stigma, Health Belief Model

## Abstract

**Background:**

Most people diagnosed with hepatitis C virus (HCV) have not linked to care, despite the availability of safe and effective treatment. We aimed to understand why people diagnosed with HCV have not pursued care in the non-urban Southern United States.

**Methods:**

We conducted a survey and semi-structured interview with participants referred to an HCV clinic who did not attend an appointment between 2014 and 2018. Our clinic is located in a non-urban region of Virginia at a university hospital. Qualitative data collection was guided by the Health Belief Model (HBM). Data was analyzed using qualitative content analysis to identify key factors influencing patient perceptions regarding HCV and pursuit of care.

**Results:**

Over half of previously referred patients (*N* = 200) could not be reached by phone. Eleven participants enrolled, including 7 men and 4 women. Based on survey responses, unreliable transportation, unstable housing, substance use, and lack of insurance were common. Participants demonstrated good knowledge of HCV disease, complications, and treatment. On qualitative analysis of semi-structured interviews, final themes emerged from within and between HBM constructs. Emerging themes influencing patient perceptions included (1) structural barriers, (2) stigma, (3) prior experiences of HCV disease and treatment, (4) discordance between the recognized severity of HCV and expected impacts on one’s own health, and (5) patient-provider relationship. Substance use was not identified to be a barrier to care.

**Conclusions:**

Participants perceived individual and structural barriers to linking to care. A strong HCV knowledge base was not sufficient to motivate pursuit of care. Efforts to improve linkage to care must address barriers at multiple levels, and system-level changes are needed. As the majority of previously referred patients could not be contacted by phone, current approaches to patient engagement are not effective for reaching these populations. Expansion of HCV care to primary care settings with an established patient-provider relationship or co-located treatment within substance use treatment programs may serve to increase access to HCV treatment.

## Introduction

The USA and the World Health Organization have established goals for elimination of hepatitis C virus (HCV) by 2030 [1, 2]. Cure of HCV improves patient-related outcomes, reduces complications related to cirrhosis and liver cancer, and prevents ongoing virus transmission [3, 4]. The HCV care cascade identifies the essential steps of HCV care necessary to achieve cure. The cascade begins with all people infected with HCV, and steps include diagnosis of affected individuals, linkage to specialty care, treatment, cure, and monitoring for reinfection [5]. The linkage to care (LTC) step requires that patients who are diagnosed are connected to a program or provider offering HCV care. In the USA, treatment has historically required specialist care, and patients must be linked from the public health or screening program where they were diagnosed to a specialty provider offering HCV treatment. National studies estimate that only 17% of people diagnosed with HCV have been linked to specialty care [6], and the current model of care is unlikely to result in HCV elimination [3]. Additionally, LTC rates may be plateauing, or even declining, following treatment of an initial group of highly motivated patients who sought care once DAAs were available [6, 7].

The rural US South has been disproportionately affected by HCV, in part due to the opioid crisis [8, 9]. HCV is highly prevalent in people who inject drugs and incarcerated populations [10, 11]. People living in the rural Appalachian region of the USA face a risk environment that may further contribute to the high burden of HCV through intergenerational poverty, geographic isolation, as well as limited employment and enrichment opportunities [12]. Poverty, homelessness, and unemployment also pose structural barriers for engagement in healthcare [13]. Among these marginalized populations, the presence of effective treatment alone is not sufficient to increase engagement with HCV care [14, 15]. This risk environment contributes to the high burden of HCV.

Varied models of care have been implemented to improve LTC, including nurse-driven care, case management, and peer support [16]. In our HCV referral clinic in the non-urban Southern United States, implementation of a nurse navigator model led to a 76% LTC rate [17]. Yet even in this high-intensity outreach model of patient education and care coordination, nearly a quarter of patients did not see a specialty provider at least 6 months following referral. The nurse navigator maintained a database including reason for not linking to care, as perceived by the nurse navigator, and the most common reasons provided included multiple no-shows to scheduled appointments, inability to contact patients to schedule an initial visit, and incarceration [17]. The reasons for missed appointments for HCV care are often complicated and diverse [18], incorporating patient-related, provider-related, and health system-related barriers [19].

Patient perceptions regarding HCV care may be changing with the availability of direct acting antivirals (DAAs), which have simplified and improved the tolerability and effectiveness of HCV treatment. The purpose of this study was to better understand why people diagnosed with HCV in the non-urban Southern United States have not pursued specialty HCV care in this era of safe and effective treatment.

To achieve our aim of understanding patient perceptions of HCV and influence of these perceptions on pursuit of HCV care, we utilized the Health Belief Model (HBM) as a guiding framework. The HBM was developed to explain engagement in health-promoting behavior as a function of six central constructs: perceived susceptibility, perceived severity, perceived benefits, perceived barriers, cues to action, and self-efficacy [20–22]. The HBM has been used to explore patient adoption of recommended care practices for asymptomatic conditions where patient beliefs may be most important in making medical decisions [23]. Given the chronic and often asymptomatic nature of HCV, this model was appropriate for our objective. Certain constructs, including perceived barriers and cues to action, allow consideration of external factors. As barriers to HCV care occur at multiple levels, inclusion of external factors is essential.

## Methods

### Clinical setting

This study took place at the University of Virginia (UVA) Infectious Diseases HCV clinic [17]. UVA is a non-urban, tertiary care, safety net hospital serving the western portion of the state of Virginia including the rural Appalachian region. Safety net hospitals care for patients regardless of ability to pay or health insurance status [24]. Referral sources include internal referrals from within the UVA health system, external referrals from community-based providers and health departments, and self-referrals. The HCV clinic staff includes physicians, a full-time nurse coordinator, and a pharmacy team. The nurse coordinator will make multiple attempts to reach referred patients through phone or mail, reschedule missed appointments, assist patients with required paperwork, complete prior authorizations, and provide general education regarding HCV over the phone. Two visits are required to receive treatment: (1) a provider visit and (2) imaging for staging of liver disease. Health insurance is not required. Through insurance coverage, with co-pay assistance if needed, and patient assistance programs, the nurse coordinator has been able to obtain medications for all eligible patients during the study time period [17].

### Study design and study population

The study population was defined to be all patients ages 18 years and older, who were referred for HCV care between November 2014 and March 2018, but who did not attend an HCV clinic visit by June 2018. This study was approved by the UVA Health Sciences Research Institutional Review Board and participants provided verbal informed consent.

The UVA Infectious Disease HCV clinic received 834 referrals for HCV treatment during this period. All patients who met the inclusion criteria (*n* = 200) were eligible for study recruitment. Interviews took place during June to August 2018 and were performed over the phone by two study personnel. Telephone recruitment was determined to be the least intrusive and only practical way to recruit participants from a cohort who did not attend their clinic appointment. Recruitment through telephone minimized barriers to participation, and telephone interviews have been demonstrated to provide rich qualitative data [25, 26]. We considered recruitment through mail or in-person, and both were deemed inappropriate. The 200 eligible patients were placed in a random order list. Consecutive sampling was used with attempts to contact all eligible patients by telephone using the random order list. The participants did not have a prior relationship with the interviewers. Research goals and the role of the interviewers in the study were explained to the participants. Participants were asked if they were in a comfortable setting where they could talk freely on the phone. Participants were given an opportunity to ask questions and to request participation at a later date, if preferred. Participation in the study required approximately 30–60 min, and participants received compensation for their time.

### Data collection

A survey and semi-structured interview were administered verbally. The survey was designed to evaluate demographic characteristics, experiences with the medical system, trust in the medical system [27], self-evaluation of overall health [28], HCV knowledge, and previously documented barriers to HCV care including unstable housing [29, 30], unreliable transportation, and history of drug and alcohol use [31, 32]. Survey questions were based on validated measurements when available, including questions regarding housing stability, self-evaluation of health, and drug and alcohol use. Knowledge questions were adapted from the literature and public health resources [2, 33, 34].

The semi-structured interview guide included questions related to each of the HBM constructs. Interviewers proceeded through the constructs in the same order for each participant and were instructed to ask clarifying questions or pursue new ideas raised by participants as indicated. Interviews were audio-recorded and transcribed verbatim. Questions explored patients’:

- *Perceived susceptibility* to complications of HCV by asking about how they feel about the impact of HCV on their health now and in the future;
- *Perceived severity* of HCV by asking about whether HCV was perceived to be a serious medical disease and any friend or family experiences with HCV complications and treatment;
- *Perceived benefits* of pursuing HCV care by asking participants about whether they felt seeking care and treatment would be effective and beneficial to their health;
- *Perceived barriers* by asking participants about what has gotten in the way of pursuing care;
- *Self-efficacy* by asking participants about how confident they are in their ability to pursue HCV care and treatment if they choose to do so;
- *Cues to action* by asking participants about what would make them more likely to pursue treatment in the future.

### Data analysis

Frequency analysis was used for survey data. Interviews were imported into Dedoose for analysis (Dedoose Version *8.0.35*, web application for managing, analyzing, and presenting qualitative and mixed method research data *(2018)*. Los Angeles, CA: SocioCultural Research Consultants, LLC www.dedoose.com). Conventional content analysis was used to identify the key factors influencing patient perceptions regarding HCV and decisions regarding pursuing care [35]. An initial codebook was developed inductively and modified as additional themes emerged from the interviews. Codes and descriptions that were applied inconsistently by study team members were revised. Each interview was coded by at least two members of the research team. Richness and quality of the data were assessed concurrently with data analysis throughout the iterative development of the themes. Thematic saturation was achieved after analysis of the first eight interviews with no additional codes required after that point. After all eleven interviews had been coded, the team grouped the codes by consensus according to the HBM constructs. If codes did not fit into a construct, a new group was created. Final themes emerged from within and between HBM categories.

## Results

### Study participants

Of the 200 patients meeting inclusion criteria, 184 had contact information available. Of these, 99 could not be reached by telephone despite up to three attempts, 64 declined participation, and 8 initially expressed interest in participation but could not find a potential time to talk with interviewers. Specific reasons for declining to participate were not elicited from those approached. Thirteen people provided consent to participate. Of these, 12 individuals completed the survey and 11 individuals completed both the survey and the interview.

Of the 11 participants who completed the entire study, the mean age was 48.2 (standard deviation 13.6) years with a range of 24 to 64 years, 7 (64%) were men, and 9 (82%) were white (Table 1). Nearly all had seen a physician in the past year, but only 6 (55%) had an established primary care provider. Over one-third were uninsured. Unstable housing and transportation were common, present for 4 (36%) and 5 (45%) of participants respectively. Five (45%) reported using drugs in the past year with 3 (27%) reporting drug use in the past month. All participants were aware of their diagnosis of HCV and that they had been referred for specialty care. Trust in medical systems was highly variable among participants. The majority of participants were referred to the HCV clinic in 2017.

**Table 1:** Participant characteristics, healthcare experiences, barriers to care, and self-reported substance use history (*N* = 11)

Characteristic	N (%)
**Demographic characteristics**	
Age group	
20–39 years	3 (27)
40–59 years	5 (45)
≥ 60 years	3 (27)
Sex	
Male	7 (64)
Female	4 (36)
Race	
White	9 (82)
Black	1 (9)
Indian American	1 (9)
Referral year	
2015	2 (18)
2016	1 (9)
2017	6 (55)
2018	3 (27)
**Healthcare experiences**	
Healthcare access	
Has an established primary care provider	6 (55)
Visited emergency room in past year	6 (55)
Has seen any doctor in past year	10 (91)
Health insurance status	
Uninsured	4 (36)
Insured	7 (64)
Private insurance	3 (27)
Medicaid	3 (37)
Medicare	1 (9)
Setting of HCV diagnosis	
Routine bloodwork by physician	5 (45)
Donating blood	2 (18)
Bloodwork while incarcerated	2 (18)
Screening at a methadone program	1 (9)
Knowledge of hepatitis C status	11 (100)
Knowledge of hepatitis C specialty referral	11 (100)
**Barriers to care**	
Unreliable transportation	
Yes	5 (45)
No	6 (55)
Unstable housing^a^	
Yes	4 (36)
No	7 (64)
Rating of own health	
Excellent	0 (0)
Very good	3 (27)
Good	4 (36)
Fair	2 (18)
Poor	2 (18)
Trust in medical system^b^, possible scores 5 to 25	
Median score [interquartile range]	18 [12–19]
Minimum score	9
Maximum score	25
**Substance use history**	
Drug use^c^	
In the past month	3 (27)
In past year	5 (45)
Alcohol Use^d^	
In the past month	2 (18)
In past year	4 (36)
Treatment for substance use disorder	
Any prior treatment	6 (55)
Alcohol	2 (18)
Drug use	3 (36)
Both	1 (9)
No prior treatment	5 (45)

*LTC* linkage to care

^a^Housing instability defined as moving 2 or more times in the past 6 months or concerned about housing stability in the upcoming 6 months [29, 30]

^b^Trust in medical system is quantified based on response to five questions and potential scores can range from 5 to 25, with higher scores indicating more trust [27]

^c^Drug screen single question, “How many times in the past month have you used an illegal drug or used a prescription medication for non-medical reasons?”, Any response ≥ 1 time is positive for drug use [31]

^d^Alcohol single question screen, How many times in the past month have you had X or more drinks in a day?, (X = 5 for men, X = 4 for women) [32]

For each of six HCV knowledge questions, at least 9 of the 11 participants answered correctly (Table 2). Each participant correctly answered over half of the questions, with 4 (36%) correctly answering four questions, 3 (27%) correctly answering five questions, and 4 (36%) correctly answering all six questions.

**Table 2:** Hepatitis C knowledge questions and response rates (*n = 11*)

Hepatitis C knowledge questions	Correct responses *N* (%)
Most people with hepatitis C do n**o**t have symptoms.	9 (82)
***True****, false*	
Most people with hepatitis C know they are infected.	9 (82)
*True,* ***false***	
A person who injected drugs one time should be tested for hepatitis C.	10 (91)
***True****, false*	
A person born between 1945 and 1965 should be tested for hepatitis C.	9 (82)
***True****, false*	
Hepatitis C can cause:	9 (82)
*Cirrhosis, liver failure, liver cancer,* ***all of the above***	
With treatment, what percent of people with hepatitis C can be cured?	9 (82)
*< 25%, 50%, 75%,* ***> 90%***	

Answer choices are in italics following question stem. Correct answer choice is in bold. Questions are adapted from the Centers for Disease Control Hepatitis C fact sheet, the World Health Organization Hepatitis C webpage, and Zeremski et al. [34]

### Qualitative analysis

The complete codebook with code descriptions, example quotes, and frequencies is presented in the Supplementary Table. While interviews were structured using the HBM, the most impactful themes arose in multiple constructs. For this reason, emerging cross-construct themes are presented here (Table 3). The key emerging themes included structural barriers to care, stigma, prior experiences with HCV through self or others, ambivalence, and patient-provider relationships.

**Table 3:** Major themes associated with pursuit of care based on qualitative patient interviews, associated Health Belief model constructs, and suggested interventions to improve care

Major themes	Associated Health Belief Model constructs	Proposed interventions to improve care
Structural barriers: financial, scheduling, transportation, health-system level	Perceived barriers	Expand Medicaid; utilize pharmaceutical company drug assistance programs; educate patients on available resources and supportive care; aim for clinic responsiveness, ease of scheduling, and confidentiality
Stigma	Perceived susceptibility Perceived barriers	Provide education on harm reduction strategies; co-locate treatment for substance use disorder and HCV; educate clinic staff on creating a welcoming atmosphere
Ambivalence	Perceived susceptibility Perceived severity	Acknowledge and address the uncertainty related to having HCV; Focus patient education campaigns on ambivalence and the potential for treatment to relieve patients of the burden of uncertainty
Prior experiences of HCV disease and treatment	Perceived susceptibility Perceived severity Perceived benefits Perceived barriers	Explore patients’ or others’ prior experiences with HCV treatment; address favorable changes in treatment since earlier therapies
Patient-provider relationship	Perceived susceptibility Perceived severity Perceived barriers Perceived benefits Self-efficacy	Encourage expansion of HCV treatment to where patients are already receiving care and have established relationships

*HCV* hepatitis C virus

### Structural barriers

Nearly all participants perceived treatment to be beneficial, yet multiple barriers prevented participants from pursuing HCV care. Common structural barriers included financial costs of treatment, unclear (or complex) referral and treatment processes, limited appointment availability, and lack of transportation. Two participants described delaying HCV care because of restrictions on HCV treatment during pregnancy. Structural barriers tended to occur in multiples, as evidenced in one gentleman’s concerns about successful follow-through with treatment: “I got money issues, transportation issues. And quite frankly, I’m a little scared to make a commitment because I don’t know whether I can honor the commitment because of my near homelessness and financial capabilities and transportation capabilities. I hate to say I’ll be here at some certain time and then I can’t find a ride, ya know. I would definitely love to pursue [HCV treatment]” [participant 10, male, 61 years].

Perceived financial barriers posed a common challenge. The perception of HCV treatment as being cost-prohibitive was based on prior experience with insurance company denials for HCV treatment, awareness of the high cost of HCV medication, or belief that insurance will not cover expensive medications. Patients perceived the cost of medication to be exorbitant, including one who expected the cost to be “thousands of dollars…It’s not like a house payment. It’s more like a whole house” [participant 7, female, 70 years].

Clinic level factors constituted barriers to care including difficulty contacting clinic staff, limited appointment availability, and gaps in time between referral and date of initial visit. Trust in the medical system and concerns regarding confidentiality were additional clinic level perceived barriers. Participants also expressed frustration with prior experiences pursuing HCV care that did not ultimately result in treatment due to prior restrictions on treatment and resulting loss of care connection. Recalling a prior visit with an HCV specialist, a participant recalled discussing treatment and “that’s all I remember, ya know, possible treatment. I don’t know if it was on my end. It just kind of fizzled out. Things never happened after that.” [Participant 6, male, 64 years]

Participants proposed modifications to the care process that would allow them to overcome barriers and pursue treatment, including changes regarding insurance/cost (*n* = 4), social support from friends and family serving as a source of motivation (3), transportation to and from appointments and required studies (2), more flexible scheduling (1), ability to easily contact doctors (1), medication side effects (1), more knowledge of the process (1), and addressing addiction (1). One participant suggested the process would be more accessible to patients if streamlined and “you could do it all at one time instead of three different appointments…It was hard enough for me to go to one” [participant 2, male, 31 years]. Four participants described their ability to overcome financial barriers by obtaining insurance through a new job or by working with clinic staff to access financial assistance programs. All participants were confident in their self-efficacy to pursue HCV treatment if they decided to do so, drawing on experiences taking medications and attending appointments for other medical problems as a source of confidence. One participant, who had attempted an earlier interferon-based regimen, reported concerns regarding side effects even for the newer, shorter course of medication: “It’s terrible what [interferon] does to you...This eight weeks crap’s got to go. That’s just shoving too much medication at one time is what it is” [participant 4, male, 51 years].

### Stigma

Stigma prevented some participants from pursuing care. Perceived stigma could be related to poverty, lack of insurance, or HCV infection and was based on prior experiences with the healthcare system. One participant who perceived stigma to be a barrier noted that “I do feel in general, the population is looked down on if you don’t have insurance or underpaid or whatever the case may be. I feel like I’m in that category, which I am.” [Participant 6, male, 64 years]. For one participant who perceived HCV-related stigma, he described his experience seeking medical care as “I go into the doctor’s office with that stigma like, here comes that dude with that hep C - everybody glove up” [participant 4, male, 51 years]. One patient who was referred to the infectious diseases HCV clinic remembered an event where “the call that came up on the caller identification at work said infectious disease clinic. At that time, I felt like I had just been labeled… I felt violated” [participant 1, female, 47 years].

Conversely, other participants did not perceive stigma to be a factor in their decision to pursue care. For these participants, experiences with healthcare led them to believe “most people in the medical field are pretty respectful people and try to understand what people are going through.” [participant 2, male, 31 years]. As another describes, “I don’t expect to be treated any differently, just as a normal patient that has a disease that needs to be cured” [participant 5, female, 29 years].

While substance use was identified as the primary risk factor for HCV infection, substance use was not perceived to be a barrier to care. One participant noted “[HCV] is a common disease. I mean, a lot of people have it. You can get it from using dirty needles. I think most of the populous believes people that have hep C got it from dirty needles, which is a stigma. But I’m not worried about that.” [Participant 10, male, 61 years]. This participant identified substance use to be beneficial to his relationship with the healthcare system because “now I can have a little access to medical services; I believe I wouldn’t have access to if I wasn’t a substance abuser” [participant 10, male, 61 years]. Concerns regarding reinfection with HCV among those with ongoing substance use did not arise.

### Ambivalence

A sense of discordance between perceived severity and perceived susceptibility emerged across interviews. Most participants perceived HCV to be a severe infection associated with complications such as liver failure, cirrhosis, and cancer as well as symptoms including fatigue, changes in mood, sleep disturbances, and jaundice. Yet, participants did not perceive that they experienced symptoms attributable to HCV, nor did they perceive themselves to be susceptible to negative outcomes.

This discordance was reflected in a participant’s answer when asked if HCV is a serious medical condition: "In general yes, I think so. In my case, I don’t know" [participant 6, male, 64 years]". Another participant explained: "for me, it’s apparently in a dormant stage. It just keeps being dormant. It hasn’t affected me. I don’t have yellow eyes or anything like that. It hasn’t noticeably affected my health…But at any time, it could jump up and affect me gravely. But so far it hasn’t." [Participant 10, male, 61 years]

Another participant expressed his knowledge of HCV: “my understanding, basically, is that it can attack your liver. It can cause cirrhosis of the liver” [participant 1, female, 47 years], while also stating “all diseases are serious. I think [HCV] is something you can live with” [participant 1, female, 47 years]. The perception of HCV as a disease that can be lived with was shared by others, including one who explained “I’m looking at my hepatitis C like prostate cancer. Men die with prostate cancer. They don’t die of it. I’m probably going to die with my hepatitis C not of it” [participant 4, male, 51 years]. Perceiving HCV to be a disease that can be safely lived with contributed to decisions not to pursue care.

### Prior experiences of HCV disease and treatment

Experience with the course of HCV disease and treatment could occur through participants' own pursuit of treatment or others'. At time of HCV diagnosis, participants found reassurance in the stories of family and friends who had been cured with DAA-based regimens, including a participant who noted: "[My husband] went through the VA Hospital and had to take treatments. And he’s been cured." [Participant 1, female, 47 years], and another who explained: "I’ve never seen anybody actually sick from it. People have told me they’ve had it. But I never met anybody who had it in an active stage [participant 10, male, 61 years]. Others who knew of family and friends’ experience with severe illness and complications related to HCV reported fear and concern on diagnosis. "I know someone who has it who was hospitalized over it. I kind of knew about it already and what to expect. Once I found out I had it, I just kind of freaked out for a minute and blocked everything out trying to deal with my own thoughts." [Participant 3, female, 24 years]

Prior experiences with interferon-based treatment led to the perception of treatment to be potentially harmful. One patient who had trialed interferon stated “[The interferon] made me feel like I was going to die, man…And that is the reason why I will not be treated - because the medication is so dangerous.” [Participant 4, male, 51 years]. Another participant, who had not sought treatment himself, described his friend’s experience with interferon: “I had a buddy of mine that was given the interferon, and it made him so weak, I had to help him from his couch to the bathroom. He used to give himself three injections a week in the stomach. I said man, I just couldn’t go through that” [participant 8, male, 55 years].

### Patient-provider relationship

The relationship between patients and healthcare providers influenced nearly all HBM constructs. Both long-standing and short-term relationships could be impactful, and the presence of trust within these relationships affected the quality of the interactions and influence on patient perceptions. Patient-provider relationships were most important when either the interaction occurred at a key step in the disease process, such as at time of diagnosis, or when based on a long-standing trusted relationship.

Receiving a diagnosis of HCV led to feelings of confusion (“where did it come from?” [participant 1, female, 47 years]), surprise (“I wasn’t sick. At least I didn’t think I was” [participant 6, male, 64 years]), and fear (“I thought I was going to die” [participant 7, female, 70 years]). Participants who interacted with healthcare providers at the time of their diagnosis cited counseling as a way to mitigate negative initial impressions. One participant reflected that “the lady that did [the test] told me that I could get treatment. It eased my mind” [participant 3, female, 24 years]. When this interaction was not present, such as for patients informed of their results through letters, feelings of bewilderment and confusion persisted.

Participants noted trusted relationships with long-standing primary care providers to be sources of motivation for pursuing treatment. One participant described how their doctor’s behavior built a trusting relationship: “I felt like my doctor was very open, honest, and straightforward. He told me about things I could do to lessen my chances of [HCV] getting worse…I say that’s the most important thing. Just be straightforward and be honest about what your prognosis is, what you need to quit doing to improve your chances, keeping from spreading it to other people. I was given all those things” [participant 7, female, 70 years]. Trusted providers were seen as potential facilitators of the treatment process, if the decision were made to seek care: “All I got to do [to get treated] is ask my family physician. My internal medicine doctor, she’s very adamant about trying to get me cured” [participant 4, male, 51 years], and “I feel like now that my doctors know everything – whatever I have going on, my doctor and I are very close. I can tell her anything. I feel like she would help me get into the clinic if I needed to rather than just me trying to do it on my own and not getting any answers.” [Participant 3, female, 24 years]

Conversely, a lack of trust with health care providers also informed patients’ decisions not to seek treatment. A patient with previous experience with earlier interferon-based treatment was discouraged by a lack of responsiveness by prior HCV specialists to his concerns, and this resulted in a lack of trust that impacted his perceived benefits of treatment. “I had taken the pill one time. And it made me sick. It made me feel like I was going to die, man. And I tried to explain it to the doctor…And the doctors just act like it ain’t no big deal…. I can’t trust [doctors] at all…It’s a serious disease. They need to treat it as one... I’d rather die from the disease than die from some cure.” [Participant 4, male, 51 years]

## Discussion

In this population of patients who have been diagnosed with HCV and referred to specialty care, but who have not successfully linked to care, excellent HCV knowledge was not sufficient for participants to pursue care. We identified themes that influenced pursuit of HCV care. The behavior of not seeking care resulted from complicated and varied factors across individual and structural levels. Dominant themes influencing pursuit of care included structural barriers to care, stigma, prior experiences with HCV through self or others, ambivalence, and patient-provider relationships. Improving LTC, and overcoming the plateau in LTC rates, requires systematic changes in care.

Structural barriers to care related to social determinants of health are prevalent. In the HBM, perceived barriers to care are one of the strongest predictors of health behavior [36]. Housing instability, unreliable transportation, drug and alcohol use, and lack of insurance have all been previously identified to be barriers to care [12–19, 37] and were common in our population. Lack of insurance and financial status were commonly perceived to be barriers, despite our clinic’s ability to access treatment for all patients, regardless of insurance status. Multiple participants hoped to overcome financial barriers by obtaining insurance to cover HCV treatment. Awareness of programs to overcome perceived financial barriers is needed among our population. Recent expansion of Medicaid, a government sponsored health insurance, in our state provides an opportunity for additional insurance coverage, which may help overcome this prominent barrier. Medicaid expansion may also provide additional resources to address transportation, another commonly cited barrier. Pregnancy arose as a barrier for two women; current HCV treatment guidelines do not recommend treatment during pregnancy [38]. Therefore clinics need to maintain a relationship with pregnant patients in order to provide access to treatment when it is appropriate in the future. Patients also raised concerns about clinic level factors including scheduling availability, responsiveness to calls, and concerns regarding confidentiality, including the possibility of inadvertent disclosure that a patient receives care at an infectious disease clinic. Limited timeframes for available appointments can impact patients’ ability to attend appointments. Simplifications to the HCV care model have been proposed, including rapid testing, test-and-treat models, minimal on-treatment monitoring, and limited patient visits, which may serve to reduce barriers, make treatment more accessible, and facilitate treatment expansion [39].

People who use drugs are a key population to target for HCV treatment [38], and the majority of our participants reported a history of substance use. Yet, in contrast to prior studies, substance use was not identified to be a barrier to care [40]. Participants reported stigma to be a factor influencing pursuit of HCV care; however, stigma was attributed to poverty, lack of insurance, or HCV infection itself, rather than to substance use. Our findings may differ from prior studies for multiple reasons. At a clinic level, our HCV clinic is co-located with a Ryan White HIV/AIDS program clinic, and clinic staff are accustomed to caring for patients with a stigmatized illness. At a policy level, our state does not have sobriety restrictions related to treatment access, though restrictions remain elsewhere in the country [41]. Among people who use drugs, HCV treatment can be a motivating factor to reduce substance use and/or to participate in harm reduction activities [42, 43]. For one participant, substance use was his main source of connection to healthcare systems. This highlights the importance of ensuring HCV treatment programs, and substance use treatment programs offer bidirectional referrals to provide comprehensive patient care, promote access to care, and potentially simplify care if services were to be co-located. A risk of HCV reinfection from ongoing substance use did not emerge as a perceived barrier to care.

Ambivalence arose as discordance between perceived severity of HCV for others relative to perceived personal susceptibility to HCV complications. People living with HCV experience a sense of “sustained uncertainty” [44]. This uncertainty can relate to the potential development of complications at an unknown future date, misinformation, or lack of knowledge regarding HCV [44, 45]. While DAAs have drastically improved the effectiveness and ease of treatment, this new era of treatment may not be influencing established patient narratives of HCV illness, limiting the uptake of new treatments [46]. To reach these patients, it may be helpful to highlight improvements in patient-related outcomes with HCV cure [3]. The most significant impact of HCV cure may be an improved sense of psychological wellbeing related to relief about no longer living with the burden of an uncertain future or the fear of transmitting the infection to others [43]. Addressing the uncertainty of living with HCV and the potential to alleviate this uncertainty through treatment may be a strategy to address ambivalence.

Prior experiences with HCV care through either personal experiences or those of family and friends are common and influenced all HBM constructs. Some patients have experienced discontinuous HCV care [47], in which patients diagnosed prior to DAA therapy have been lost to HCV care. Experience with interferon-based treatment regimens can motivate some patients to seek the more patient-friendly DAA treatment [48], though in our study, the severe side effects associated with interferon persist in patients’ memories and discourage them from reconnecting to care. People living with HCV have varied reasons for not pursuing care, and providers should explore patient perceptions, including knowledge of others’ experiences with HCV treatment, and an individual’s own prior experiences.

Established patient-provider relationships influence perceived barriers and self-efficacy. The quality of these patient-provider relationships determines if they serve as barriers or facilitators to care. Positive patient-provider relationships are beneficial in HCV treatment [48, 49]. Multiple patients cited their trusted provider as a key source of motivation for pursuing treatment and someone they could turn to for assistance. As most participants had seen a doctor in the prior year, HCV treatment could build on established relationships by expanding to sites where patients are already receiving care such as primary care practices, health departments, or substance use disorder treatment programs [50]. Expansion into other sites of care may also serve to decrease logistical barriers and decrease stigma. Yet, primary care providers remain hesitant to treat HCV, and HCV providers hesitate to treat those with substance use, resulting in limited uptake of these models of care [51–53].

Notable limitations of our study include inability to contact a majority of those who did not link to care in our clinic. Low participation may have been due to recruiting over the telephone, which is a communication method through which it can be harder for a researcher who is unknown to the participant to build rapport. Patients who could not be contacted may have different experiences living with HCV, including unstable phone numbers, which likely impacts their experience seeking care. Additionally, many of those contacted declined participation, and this may be related to prior experiences with the healthcare system, competing priorities, or other factors. Eligible participants may have been referred to the clinic over a year prior to the time of recruitment, and over that time patients may have changed phone numbers or forgotten about the referral, limiting recruitment. Our data is limited to the UVA health system, and eligible participants may have sought outside HCV care. Although our sample size was small, we were able to achieve thematic saturation in our analysis of the interviews, which suggests that the sample was adequate to elicit a consistent set of themes. The analysis revealed barriers to care linkage experienced by interview respondents, related to underlying social determinants of health. Generalizability of our findings is limited by our clinic structure. Use of a nurse-navigator model likely contributed to participants’ excellent HCV knowledge, as the nurse provides education over the phone while discussing the logistics of referral. However, not all clinics have a dedicated full-time nurse coordinator able to provide this level of education and coordination. While our study was initially designed using the HBM to structure interviews, we found limitations of this model. The model focuses on the individual, and the HBM constructs do not directly account for social, environmental, or emotional factors that impact health behaviors. During qualitative interviews, structural barriers arose as a key factor in perceptions of HCV care. For this reason, we performed our analysis using qualitative content analysis to identify higher level themes that emerged across constructs.

## Conclusions

Basic knowledge about HCV was important, but not sufficient, for patients to pursue care as barriers prevent patients from acting on this knowledge. Barriers occur at the individual and structural level, and nearly half of eligible patients could not be contacted demonstrating that current care methods, especially telephone coordination, are not sufficient to reach a large proportion of people with HCV who could benefit from treatment. Overcoming the recent plateau in LTC rates will require changes in models of care. On an individual level, public health campaigns should focus on the ease of treatment, address the uncertainty that arises regarding how hepatitis C will affect one’s own health, and emphasize potential improvements in patient-related outcomes with treatment. Expanding access to health insurance may overcome perceived financial barriers. Providing HCV care in settings with an established patient-provider relationship, or in conjunction with substance use treatment programs, may increase treatment uptake.

## Supplementary information

**Additional file 1:** **Table S1.** Study codebook including codes, definitions, and example quotes with frequency of occurrence.

## Data Availability

The datasets generated and/or analyzed during the current study are not publicly available due to the sensitive and personal nature of qualitative interviews but are available from the corresponding author on reasonable request.

## Abbreviations

HCV: Hepatitis C virus
DAAs: Direct-acting antivirals
LTC: Linkage to care
UVA: University of Virginia
HBM: Health Belief Model
