# Supplementary material for: A qualitative study of perceived barriers to hepatitis C care among people who did not attend appointments in the non-urban US South
Source: Harm Reduct J. 2020 Sep 18;17:64. doi: 10.1186/s12954-020-00409-9 (PMC7501689; doi:10.1186/s12954-020-00409-9)
Supplement: Supplementary file 1 — Additional file 1: Table S1. Study codebook including codes, definitions, and example quotes with frequency of occurrence. [file 12954_2020_409_MOESM1_ESM.docx]

**Supplementary Table 1:** Study codebook including codes, definitions, and example quotes with frequency of occurrence.

| Code | Code Definition | Example Quote | N (%) |
| --- | --- | --- | --- |
| Diagnosis Experience | | | |
| Testing Circumstances | Describes the circumstances regarding why a person was tested for hepatitis C | “The first time I found out or was told I had it I guess was after a friend talked me into donating blood or donating plasma or something like that at some blood bank. When you do that, I guess they test your blood for different diseases.” [Participant 2, male, 31 years] | 11 (100) |
| Feelings at time of Diagnosis | Describes participant's initial thoughts and feelings at time they received the diagnosis of hepatitis C | “I truly didn’t know what to think. I was very upset. My wife left me over it. She got tested. She come up negative. We ain’t together today because of it. I lost my children and all.” [Participant 11, male, 46 years] | 11 (100) |
| Method of communication of diagnosis | Describes how the diagnosis was communicated to the patient | “They sent me a piece of paper. And they said for me to take it to my doctor. I took it to my doctor and she said – hep C I guess I had, not B – and we should run some tests.” [Participant 7, female, 70 years] | 11 (100) |
| Counseling provided at time of diagnosis | Patient’s recollection of the counseling or information they received regarding hepatitis C at the time of diagnosis | Interviewer: When you were diagnosed with hepatitis C, what information were you given about the disease?  Interviewee: “Well, I think I might’ve gotten a packet or two and told it affects your liver.” [Participant 2, male, 31 years] | 10 (91) |
| Referral Experience | | | |
| Referral Experience | Describes the patient’s experience of the referral process including the circumstances | “I think somewhere along the line, I think I maybe got referred. I’ve actually been seen twice. The second time I was referred – when I mentioned earlier about a possibility of treatment – I went to the hospital; I was having palpitations. I thought I was having a heart attack, but it turned out otherwise. And then one thing led to another.” [Participant 6, male, 64 years] | 10 (91) |
| Previously attended a hepatitis C specialty visit | Describes experience with hepatitis C specialty care prior to referral to our clinic | “I’ve been to four [Hep C specialists] so far. And there ain’t none of them done a damn thing for me.” [Participant 4, male, 51 years] | 4 (36) |
| Expectations of referral or treatment process | Describes patient expectations for future experiences with the referral and treatment process. This is the default code for comments referring to the referral/treatment process, unless comment specifically refers to expectations regarding the provider. | “From my understanding, they do another blood draw to see exactly where my levels are and everything to see what dose of the medication they were to give me. I believe it’s an eight-or twelve-week program. The medication for it is curable.” [Participant 5, female, 29 years] | 9 (82) |
| Perceived Susceptibility | | | |
| Risk Factor - Substance Use | Describes participant's perception of substance use as a risk factor and any descriptions of their history of substance use | “I knew I already had it because like I said, I used a lot of needles after not just one person – probably 50 people. It wasn’t good.” [Participant 8, male, 55 years] | 8 (73) |
| Risk Factor - Sexual Activity | Describes participant perception of past sexual activity as a risk factor for hepatitis C | “My original thoughts and feelings were where did it come from? It was just the fact of where is this coming from. Reading up on it online, I could see that it could be sexually transmitted.” [Participant 1, female, 47 years] | 2 (18) |
| Disease that can be lived with safely | Describes participant perceptions that hepatitis C can be lived with safely, without complications | “All diseases are serious. I think [Hep C] is something you can live with…I just don’t believe it was probably dormant and was there this whole time, and I’ve been fine all this time. That’s why I think it’s something you can live with.” [Participant 1, female, 47 years] | 2 (18) |
| Impact on lifestyle | Describes perceptions on how hepatitis C impacts participant's lifestyle | “Most of my understanding is I have to take care of myself. I have to eat right, no alcohol, no drugs, no Tylenol, no Acetaminophen – basically take good care of myself” [Participant 11, male, 46 years] | 2 (18) |
| Perceived Severity | | | |
| Limited Knowledge or Understanding | Patients identify a lack of knowledge regarding hepatitis C disease, severity, or potential complications | “I’m not real clear on it. That’s my problem, too. All the doctors tell me is this is going to affect your liver 30 years from now, or what have ya. I mean, what the hell. Is that all you can tell me? I don’t know anything about this hepatitis C. And I still don’t. They never explained to me what hepatitis C virus does or what it does or anything.” [Participant 4, male, 51 years] | 3 (27) |
| Ambivalence | Patients have an understanding of hepatitis C, and perceptions of severity include mixed feelings regarding the severity of disease, or regarding severity of disease for others versus impact on self | “For me, it’s apparently in a dormant stage. It just keeps being dormant. It hasn’t affected me. I don’t have yellow eyes or anything like that. It hasn’t noticeably affected my health. I don’t understand it. But apparently, it’s in a dormant phase. But at any time, it could jump up and affect me gravely. But so far it hasn’t” [Participant 10, male, 61 years] | 7 (64) |
| Complications/Severe disease | Described perceptions of medical issues that may arise in the future due to hepatitis C or hepatitis C as a severe disease | “You don’t get it under control, taken care of, it’ll kill ya… it can cause cancer, cirrhosis, yellow jaundice. You get all that and liver failure.” [Participant 8, male, 55 years] | 10 (91) |
| Symptoms | Describes the presence of symptoms that patient attributes to hepatitis C | “You lose a lot of weight when you got hep C… It does affect your appetite. It affects your sleep. It affects your mood.” [Participant 4, male, 51 years] | 2 (18) |
| Other people’s experiences | Perceptions regarding family members’ and friends’ experience with hepatitis C infection, or lack of knowledge of other people's experiences with hepatitis C | “My brother had hepatitis C. I could’ve even got it from him using needles – each other, me and him. He had it for probably 20 years before he even knew he had it” [Participant 8, male, 55 years] | 10 (91) |
| Viral Clearance | Perception that viral clearance has happened or that patient expects it to happen, and does not perceive a need to pursue further care | “It had cleared up. I’m supposed to be tested again in a couple months just to be sure. But [the doctor] said it had kind of cleared up on its own.” [Participant 3, female, 24 years] | 2 (18) |
| Perceived Benefits | | | |
| Knowledge of treatment availability and effectiveness | Describes patient awareness of the availability of treatment for hepatitis C and/or describes knowledge of effectiveness of treatment in curing disease | “Really the only thing that I know about it is that it’s a 90-day treatment, and you take one pill every day, I think. And after that, it’s supposed to clear up. That’s really all I know about it.” [Participant 3, female, 24 years] | 10 (91) |
| New medications compared to old | Describes perceptions regarding the availability of new medications that have an improved side effect profile and cure rates compared to older medications | “I knew about the interferon. I had a buddy of mine that was given the interferon, and it made him so weak, I had to help him from his couch to the bathroom. He used to give himself three injections a week in the stomach. I said man, I just couldn’t go through that…Now I heard about this knew pill that came out. And it says it’s supposed to be 99.9 or even 100 percent curable.” [Participant 8, male, 55 years] | 3 (27) |
| Perceived Barriers | | | |
| Limited Knowledge | Describes limited knowledge of treatment or the referral process that patient perceives to be a barrier to further pursuit of care | “I know they go this new drug that’s a 12-15-week treatment or something where ya take a pill a day or a pill every week or something. I can’t recall. It’s been a while since I spoke with anybody. I know really very little other than these commercials I’ve seen on TV.” [Participant 6, male, 64 years] | 5 (45) |
| Logistical - Transportation | Describes transportation as a logistical barrier preventing linkage to care | “I don’t have a vehicle. I have no driver’s license. I don’t have anybody like family to take me” [Participant 11, male, 46 years] | 3 (27) |
| Logistical - Scheduling or Work | Describes work or scheduling conflicts as logistical barriers preventing linkage to care | “The last time I was supposed to go work got in the way. And I should have gotten off and come back home to go. But I was kind of bouncing around at the time. I think I made the appointment six weeks in advance; that was how long it took to get in there. And then I didn’t realize it was the same week that I was working out of town. And I just never made another appointment after that.” [Participant 2, male, 31 years] | 6 (55) |
| Logistical - Pregnancy | Describes pregnancy as a logistical barrier preventing linkage to care | “They referred me to the infectious disease doctor through UVA. It made it fairly easy to get in and be seen. I just kind of had some issues with work and scheduling and stuff like that. And the fact that I’m pregnant, they don’t like to do treatments unless my blood levels show that I need it right then and there.” [Participant 5, female, 29 years] | 2 (18) |
| Financial | Describes financial barriers preventing linkage to care includes concerns regarding cost of treatment, insurance status, and insurance coverage of treatment. | “My understanding is this medication is thousands – and I’m not talking two, three, ten, and fifteen thousand. I’m talking major thousands of dollars if somebody has to pay for it out of their pocket. It’s not like a house payment. It’s more like a whole house. That’s ridiculous.” [Participant 7, female, 70 years] | 8 (73) |
| Substance Use Disorder | Describes substance use disorder as a barrier to care. | Interviewer: Do you think your history of or active substance use will prevent you from receiving a prescription for hepatitis C medication?  Participant: I don’t know. I can’t answer that. It’s possible. [Participant 11, male, 46 years] | 1 (9) |
| Stigma | Describes stigma as a barrier to care. | “I go into the doctor’s office with that stigma like, here comes that dude with that hep C –everybody glove up and everything.” [Participant 4, male, 51 years] | 4 (36) |
| Trust in Medical System | Describes trust in the medical system as a barrier to care, including concerns regarding trust in providers or maintenance of confidentiality as a barrier to care. | “[I] received a phone call at my office that the appointment had been cancelled. I got a phone call from a coworker that told me that…And I was just very distraught, I guess, with she informed me that the information was left her, No. 1. And the call that came up on the call ID at work said infectious disease. At that time, I felt like I had just been labeled…I guess it probably was more so how she came across with saying an infectious disease clinic called. I don’t know how to explain it. I felt like I was violated…if that makes sense. I felt violated.” [Participant 1, female, 47 years] | 3 (27) |
| Self-Efficacy | | | |
| Confidence in Self - General Confidence | Describes participant’s general confidence in their ability to complete the necessary steps to achieve cure | “I got money issues, transportation issues. And quite frankly, I’m a little scared to make a commitment because I don’t know whether I can honor the commitment because of my near homelessness and financial capabilities and transportation capabilities. I hate to say I’ll be here at some certain time and then I can’t find a ride, ya know. I would definitely love to pursue it.” [Participant 10, male, 61 years] | 9 (82) |
| Confidence in Self - Ability to complete clinic requirements make appointment | Describes ability to make and attend an appointment and/or complete required labs/imaging and paperwork | Interviewer: How confident are you that you could complete necessary steps to receive treatment such as blood tests, liver imaging, paperwork, and follow-up visits?  Participant: Fairly good. Like I said, I followed up on all my cancer problems, going to Charlottesville and being there on time and everything else. I could do it if I had the means. [Participant 10, male, 61 years] | 10 (91) |
| Confidence in Self - Ability to take medications | Describes participant’s confidence in their ability to take medications as prescribed | “If I wasn’t pregnant, I’m confident that I could take a pill every day. I take prenatals every day.” [Participant 3, female, 24 years] | 11 (100) |
| Ability to overcome barriers for self | Describes participants’ perception of their ability to overcome potential barriers | “The medication is expensive. But with some programs and everything, it’s fairly reasonably priced. And if you the correct insurance that’s free and it’s an eight-to twelve-week program – and it usually comes in pill form.” [Participant 5, female, 29 years] | 6 (55) |
| Absence of logistical barriers | Describes perception that a proposed logistical barrier does not apply to this individual | “Right at the present, I’m not working. But even if I was and I had a doctor appointment, I have no problems at all about missing a doctor’s appointment.” [Participant 6, male, 64 years] | 4 (36) |
| Absence of financial barriers | Describes perception that a proposed barrier, financial concerns, does not apply to this individual | Interviewer: Did you have any issues with your insurance covering the cost of your treatment?  Participant: We haven’t began any treatments. But insurance has been good so far. [Participant 9, male, 63 years] | 2 (18) |
| Absence of stigma as a barrier | Describes perception that a proposed barrier, stigma, does not apply to this individual | “I don’t expect to be treated any differently, just as a normal patient that has a disease that needs to be cured” [Participant 5, female, 29 years] | 8 (73) |
| Absence of substance use disorder as a barrier | Describes perception that a proposed barrier, substance use disorder, does not apply to this individual | Interviewer: How does substance use affect your relationship with the healthcare system?  Participant: Actually, it makes it better.  Interviewer: Okay, interesting.  Participant: Now I can have a little access to medical services, I believe I wouldn’t have access to if I wasn’t a substance abuser. [Participant 10, male, 61 years] | 3 (27) |
| Trust in health systems as a source of support | Describes perception that health systems or medical providers are a facilitator to care | Interviewer: And how much do you think you can trust the hepatitis C doctor?  Participant: Very much so. [Participant 11, male, 46 years] | 4 (36) |
| Modifications to process that would be needed to overcome barriers | Described changes that would need to occur in the process of linking to care, being evaluated and treated for hepatitis C or changes in patient circumstances that would enable them to overcome barrier. Conditional situations that would improve the likelihood patient pursues care are included in this section. | “I think my biggest concern because I don’t like making bills, if I could get an overview of how many treatments I would need and I knew what I was dealing with up front so I could fit that in my budget to do, then yeah, I would probably pursue [treatment].” [Participant 1, female, 47 years] | 8 (73) |
| Knowledge of Process | Describes ability of participant to gain knowledge regarding the referral or treatment process | “I think I pretty much know how that would work. I know they want ya to quit drinking and this and that and take your meds regularly…I pretty much know the ropes and standard procedure.” [Participant 6, male, 64 years] | 3 (27) |
| Cues To Action | | | |
| Importance of seeing a specialist | Describes participant's perception regarding importance of attending an appointment with a hepatitis C specialist, including perceptions that suggest this step is or is not important | Interviewer: Do you feel seeing a hepatitis C doctor is important to your health?  Participant: “Oh, yeah.” [Participant 11, male, 46 years] | 7 (64) |
| Social Support | Identification of social support as a cue to action | “I have a very strong support system. I count myself unusually lucky in the people that I have around me.” [Participant 7, female, 70 years] | 2 (18) |
| Source of Motivation | Describes participant identified sources of motivation | “I’d like to get it cleared up. I’m all for trying to have a healthy body.” [Participant 10, male, 61 years] | 3 (27) |
| Patient Provider Relationship | | | |
| Past/Current Experiences with medical system | Describes perceptions of past or current experiences with the medical system in general | “Whatever I have going on, my doctor and I are very close. I can tell her anything.” [Participant 3, female, 24 years] | 11 (100) |
| Expectations Regarding Future Experiences | Describes expectations regarding the patient/provider relationship within the context of Hep C care, including past experiences that form the basis for this expectations | “I think I could trust a hep C specialist.” [Participant 2, male, 31 years] | 7 (64) |
